# Targeting Mitochondria during Cold Storage to Maintain Proteasome Function and Improve Renal Outcome after Transplantation

**DOI:** 10.3390/ijms21103506

**Published:** 2020-05-15

**Authors:** Sorena B. Lo, Richard T. Blaszak, Nirmala Parajuli

**Affiliations:** 1 Department of Pharmacology and Toxicology, University of Arkansas for Medical Sciences, Little Rock, AR 72205, USA; SBLo@uams.edu; 2 Division of Nephrology, Department of Pediatrics, University of Arkansas for Medical Sciences, Little Rock, AR 72205, USA; BlaszakRichardT@uams.edu

**Keywords:** renal cold storage and transplantation, mitochondrial function, proteasome function, therapeutics

## Abstract

Kidney transplantation is the preferred treatment for end-stage kidney disease (ESKD). Compared to maintenance dialysis, kidney transplantation results in improved patient survival and quality of life. Kidneys from living donors perform best; however, many patients with ESKD depend on kidneys from deceased donors. After procurement, donor kidneys are placed in a cold-storage solution until a suitable recipient is located. Sadly, prolonged cold storage times are associated with inferior transplant outcomes; therefore, in most situations when considering donor kidneys, long cold-storage times are avoided. The identification of novel mechanisms of cold-storage-related renal damage will lead to the development of new therapeutic strategies for preserving donor kidneys; to date, these mechanisms remain poorly understood. In this review, we discuss the importance of mitochondrial and proteasome function, protein homeostasis, and renal recovery during stress from cold storage plus transplantation. Additionally, we discuss novel targets for therapeutic intervention to improve renal outcomes.

## 1. Introduction

Acute kidney injury affects approximately 13–18% of hospitalized patients and has been shown to be associated with increased mortality. [1]. Additionally, acute kidney injury has also been linked to the development of chronic kidney disease and end-stage kidney disease (ESKD). ESKD affects 630,000 Americans and is the ninth-leading cause of death in the US (NIDDK, 2014). Kidney transplantation is the preferred treatment to increase longevity and quality of life for people with ESKD, but due to a shortage of transplantable kidneys, 7 of 10 ESKD patients will remain on dialysis (and many will die) while waiting for a kidney (95,268 waiting-list candidates vs. 19,848 transplants in 2017; http://www.unos.org).

Advances in tissue-type matching and immunosuppressive protocols have greatly reduced the incidence of acute transplant rejection and short-term graft dysfunction; however, optimizing long-term graft function continues to be a challenge, especially with kidneys from deceased donors. Kidneys from living donors have better long-term graft outcomes than those from deceased donors. One of the key variables is cold storage (CS) [2,3,4], which is the universal method for preserving kidneys from donors that allows time for identification of potential recipients, transportation of kidneys, tissue typing, and cross-matching. Kidneys from living donors generally are exposed to only a few hours of CS, while those from deceased donors undergo long hours of CS during transport, tissue typing, and cross-matching. Despite the increasing use of hypothermic machine perfusion (HMP) for the preservation of deceased donor kidneys, most transplant centers still rely on static CS techniques. Both CS techniques (HMP or static) lower the metabolic rate, allowing the organ to be stored until a recipient is located [5,6]. Acceptable CS times vary between transplant centers, ranging from 24 to 72 h. Unfortunately, each additional hour of CS increases the risk of graft failure [2,3,4] via poorly described mechanisms. Tragically, approximately 20% of donor kidneys that are retrieved each year are discarded or not transplanted (http://www.unos.org), partly due to prolonged CS [7,8]. A better understanding of injury-related pathways secondary to CS, and the identification of novel therapies aimed to mitigate damage would likely lead to improved deceased donor transplant rates and patient outcomes.

The kidney is a composite organ made up of many blood-filtering units called nephrons. Each nephron consists of anatomically and functionally discrete segments known as a renal corpuscle, proximal tubule, loop of Henle, distal tubule, and collecting duct system [9]. Each segment of the nephron is composed of multiple cell types of both epithelial and mesenchymal origin; all of which participate in various functions such as removing nitrogen waste and other waste products, controlling blood electrolytes and acid-base balance, and secreting hormones that regulate blood composition and blood pressure [9,10,11,12,13]. Like most organs, renal tissues consume a high level of oxygen [14,15,16], and have quality-control mechanisms that help fold nascent polypeptides, clear unfolded or misfolded proteins, respond to protein aggregates, and dispose of potentially toxic molecules. The balance of energy and the integrity of the proteome is extremely important for renal cell viability during stressful conditions, especially during renal cold storage plus transplantation (CS/Tx). One could reasonably expect to see damage within various compartments of renal tissue when the kidney undergoes a series of stressors, namely CS and blood reperfusion events during transplantation. Recovery from CS/Tx-induced stress is possible when the repair process remains in balance and overcomes the rate of CS/Tx-mediated injury. Unfortunately, very little is known about the mechanisms of CS/Tx-mediated damage that leads to renal dysfunction after CS/Tx. Here, we will discuss some of the literature that describes the triggers of damage during renal transplantation. Specifically, we will discuss the importance of mitochondrial and proteasomal function to maintain mitochondrial protein quality during renal CS/Tx and potential strategies to prevent organ damage and improve transplant outcomes.

### 1.1. Ischemia/Reperfusion Injury

The early events of injury in the deceased-donor kidney start with ischemia, wherein the tissues are deprived of blood, and thus oxygen and nutrients. Ischemia increases a number of intracellular changes within renal cells, including protein denaturation [17]. Donor kidneys are further exposed to cold ischemia during CS. During transplantation, vascular anastomosis followed by blood reperfusion introduces warm ischemia and reperfusion, also known as ischemia–reperfusion injury (IRI). IRI is an unavoidable event during deceased donor kidney transplantation and impacts short-term and long-term graft outcomes [18]. Indeed, the experimental models [19,20,21] and a clinical report of CS/Tx [2] showed that severe renal injury is produced by prolonged CS. Hence, it is important to understand the pathophysiological mechanisms of IRI-mediated renal injury following CS/Tx.

**Figure 1:**
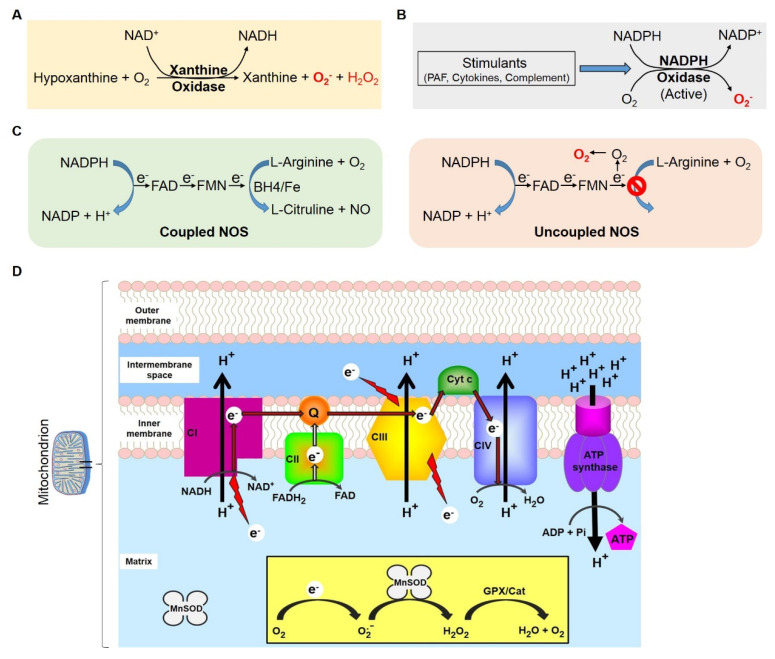
Mechanism of reactive oxygen species production. (**A**) Xanthine oxidase. During ischemic conditions, cellular ATP is actively broken down into hypoxanthine. With the presence of molecular oxygen following reperfusion, hypoxanthine (a product of ischemia) is catalyzed by xanthine oxidase via an oxidation reaction to produce xanthine, superoxide and hydrogen peroxide. (**B**) Nicotinamide adenine dinucleotide phosphate (NADPH) oxidase. During IRI, several stimulators such as cytokines, platelet-activating factor (PAF), and complement proteins activate NADPH oxidase, which donates one electron to molecular oxygen and produces a superoxide radical. (**C**) Nitric oxide synthase (NOS). The reductase domain of NOS contains flavins (flavin mononucleotide (FMN) and flavin adenine dinucleotide (FAD)) and binds NADPH, whereas the oxygenase domain contains heme and tetrahydrobiopterin (BH_4_) and this domain binds L-arginine. Heme in the presence of BH_4_ allows the transfer of electrons from the reductase domain to the oxidase domain, therefore allowing for O_2_ reduction and formation of nitric oxide (NO). During IRI this coupling is interrupted, which favors superoxide production. (**D**) Mitochondrial electron transport chain. Five mitochondrial respiratory complexes (complexes CI-CV, CV is also known as ATP synthase) are involved in the oxidative phosphorylation process in which the electrons are transferred from CI and CII to CIII and CIV. CIV is a terminal electron acceptor and converts molecular oxygen to water. The protons are pumped to the inner membrane space, which is then used by ATP synthase to generate ATP. During electron transfer, some electrons are leaked and form superoxide, which is scavenged by the mitochondrial antioxidant, manganese superoxide dismutase (MnSOD), and finally the ROS is detoxified by glutathione peroxidase (GPX) and catalase (Cat).

IRI produces a burst of reactive oxygen species (ROS) through different mechanisms, mostly via aberrant regulation or activation of enzyme systems. The enzyme systems that are commonly implicated in ROS production in post-ischemic tissues are xanthine oxidase, nicotinamide adenine dinucleotide phosphate (NADPH) oxidase, uncoupled nitric oxide synthase (NOS), and mitochondrial respiratory complexes, also known as electron transport chain (ETC) (Figure 1). Xanthine oxidase is a potential source of ROS during IRI. During ischemic insult, this enzyme is generated from xanthine dehydrogenase by limited proteolysis or by sulfhydryl oxidation [22]. Ischemic injury leads to the catabolism of adenosine triphosphate (ATP) and subsequent accumulation of hypoxanthine [23]. Following reperfusion, in the presence of molecular oxygen, xanthine oxidase catalyzes hypoxanthine to produce xanthine and ROS (superoxide, O_2_^−^, and hydrogen peroxide) (Figure 1A and [24,25,26]). Another well studied enzyme system that plays a critical role in producing ROS is NADPH oxidase, which is ubiquitously expressed in kidneys [27]. Renal IRI leads to production of a variety of chemical mediators (e.g., cytokines, complements, and platelet activation factor) that can increase the expression and activity of NADPH oxidase [28,29,30]. When activated, NADPH oxidase reacts with molecular oxygen post-IRI and produces superoxide and downstream oxygen radicals (Figure 1B and [31]). The third enzyme system to produce ROS is NOS. Typically, 3 forms of NOS (endothelial, neuronal, and inducible) are expressed in mammalian cells and are available in monomer and homodimer states [32]. NOS isoforms have both a reductase and an oxygenase domain. The reductase domain contains flavins (flavin mononucleotide (FMN) and flavin adenine dinucleotide (FAD)) that bind to NADPH. The oxygenase domain contains heme and tetrahydrobiopterin (BH_4_) and binds L-arginine [21]. BH_4_ can only bind to the NOS dimers and is therefore a key factor in coupling NOS. Monomeric NOS transfers electrons from NADPH to flavin and a limited amount of superoxide is produced during this process [32,33]. NOS dimers are stable and couple oxygen reduction to the synthesis of nitric oxide via oxidation of L-arginine (substrate) in the presence of co-factors (e.g., BH_4_) (Figure 1C and [32,33]). During IRI, dimer disassembly occurs and the ratio of dimer/monomer NOS falls, which results in uncoupling of NOS and thereby the production of superoxide and downstream oxygen radicals (Figure 1C [34,35]). Another source of cellular ROS is the mitochondrial respiratory complex or ETC (Figure 1D), discussed in the mitochondrial injury section below. Regardless of the source, renal CS/Tx displays an increase of ROS production [23,36,37,38]. Elevated ROS can further trigger several pathological effects, including protein denaturation, mitochondrial calcium overload, and elevated phosphate concentrations, all of which lead to renal damage [37,39]. 

Oxygenation of renal tissue remains critical for its survival. Under normal conditions with proper blood supply, renal tissue experiences an excess of molecular oxygen. The cessation of supplied oxygen during ischemia promotes renal tissue hypoxia, which becomes more severe during IRI. Normally, hypoxia stimulates a protective cellular response by activating a critical transcription factor, hypoxia-inducible factor (HIF) [40]. HIF is composed of two subunits, namely the HIFα subunit and the constitutively expressed HIFβ subunit [41]. At least 3 isoforms of the HIFα subunit have been identified; 2 of these are widely studied in the kidney, namely, HIF-1α, which is expressed in tubular epithelial cells, and HIF-2α, which is expressed in interstitial, endothelial and fibroblastic cells [42]. Under normoxic conditions, HIF-1α is hydroxylated by prolyl hydroxylase (PHDs) enzymes [43]. The hydroxylated form of HIF1α binds to the von-Hippel-Lindau tumor suppressor protein (pVHL) as a part of the HIF-1α/pVHL/ubiquitin ligase complex [44,45]. This complex formation leads to ubiquitination of HIF-1α and degradation of this protein by the ubiquitin-proteasome system (UPS), and therefore the level of HIF-1α remains low [44]. However, under hypoxic conditions, the prolyl hydroxylation of HIF-1α is suppressed and this protein escapes UPS degradation and localizes into the nucleus, where it forms a dimer with HIF-1β. This HIF-1α/HIF-1β dimer regulates the induction of several genes that mediate metabolic adaptation, angiogenesis, erythropoiesis, cell growth, survival, and apoptosis [46,47]. For this reason, the HIF-1α pathway has been an attractive drug target to ameliorate IRI. HIF-1α signaling can be upregulated prior to IRI by ischemic preconditioning (local or remote) of the organ or by using HIF mimetic agents; both approaches have mitigated IRI and improved organ function in pre-clinical small animal models, but have all failed in large animal models and human trials [48]. Interestingly, chronic hypoxia appears to be detrimental to long-term renal viability after transplantation [49]. Adequate reperfusion in the graft is needed for renal function recovery by reducing hypoxic tissue injury. Although reperfusion restores molecular oxygen, the vascular damage during IRI leads to a reduction in renal blood flow of up to 50% after reperfusion [50,51,52,53]. This partial blood flow or no-reflow phenomenon limits the oxygen supply that is much-needed by the renal tubules to achieve solute exchange [54] and a high rate of aerobic glycolysis [55]. It has been shown that the level of tubulointerstitial damage determines long-term graft outcome, and it has been further proposed that chronic hypoxia in the tubulointerstitium is a common pathway for renal failure after CS/Tx [56]. Hypoxic injury during IRI releases cellular factors, such as cytokines, chemokines and apoptotic/necrotic bodies. These factors activate innate and adaptive immune systems [57,58,59,60]. This inflammatory response is needed to clear the injured tissue; however, the overwhelmed immune response often leads to tissue destruction. Targeting the factors that initiate inflammation may be more effective than targeting downstream effectors. Similarly, inhibition of a given pathway early in the course of injury may be beneficial but could bring harmful results if performed later in the resolution phase of injury [61,62,63,64]. It has been suggested that the inhibition of the toxic or inflammatory factors be performed at the stages of donor kidney CS to successfully prevent reperfusion mediated injury following transplantation.

### 1.2. Mitochondrial Injury

Mitochondria are unique organelles that have their own genome. Mitochondrial DNA (mtDNA) is a double-stranded circular DNA of approximately 16.5 kb that contains 37 genes that transcribe 13 messenger RNAs, 22 transfer RNAs and 2 ribosomal RNAs. These genes encode 13 subunits of the mitochondrial respiratory complexes (I, III, IV and V). The other mitochondrial proteins are encoded in the nuclear genome and are synthesized in the cytosol and imported into mitochondria. It has been recognized recently that mitochondria contribute to the pathogenesis of acute and chronic kidney injury via several mechanisms, which are discussed below. 

#### 1.2.1. Fission and Fusion

Mitochondria are highly dynamic organelles that undergo constant fission (division and production of short mitochondria) and fusion (the mixing of two or more mitochondria and production of a long filamentous morphology) to maintain overall mitochondrial health [65]. Fusion is needed to overcome the stress by the mixing of partially damaged mitochondria with complementation by healthy mitochondria. Fission helps to increase the number of mitochondria (mitochondrial biogenesis) as well as to rid cells of mitochondria damaged by stress. Dynamin superfamily members, such as Opa1, and mitofusin 1 (MFN1) and 2 (MFN2) are large GTPases that mediate fusion in eukaryotic cells. Opa1 is involved in inner mitochondrial membrane fusion and MFN1 and MFN2 are involved in outer mitochondrial membrane fusion. Fission is mediated mainly by dynamin-related protein 1 (Drp1). Mitochondrial fission and fusion are required for mitochondrial inheritance and maintenance of mitochondrial function as these processes help to segregate damaged mitochondrial contents during stress. Interruption of these processes could lead to the pathogenesis of numerous diseases, including acute kidney injury. Several reports support the notion that warm IRI leads to significant changes in mitochondrial dynamics, morphology, and function [66,67,68,69,70,71]. Mitochondrial fission is a significant contributor to renal IRI and Drp1 plays a pivotal role in this process during renal IRI [66,67,72]. Brooks et al. demonstrated that Drp1 localizes to mitochondria early during IRI, leading to fragmentation of mitochondria in renal tubules [66]. Similarly, Li et al. have shown that phosphorylation of Drp1 by mammalian STE-20-like kinase 1 leads to mitochondrial fission, which subsequently contributes to renal IRI [67]. Furthermore, pharmacological inhibition of fission, achieved by inhibiting Drp1 function, reduced mitochondrial fragmentation and improved renal function following renal IRI [66]. On the other hand, mitochondrial fusion plays a pivotal role in maintaining mitochondrial health/function. Opa1 is a critical protein involved in mitochondrial fusion and is needed for the maintenance of mitochondrial fusion and mitigation of injury during stress. It appears that Opa1 is maintained via signaling involving sirtuin3 and extracellular signal-regulated kinase [73]. Wang et al. revealed that renal IRI-mediated down regulation of sirtuin3 leads to perturbation of mitochondrial fusion via Opa1 proteolysis [73]. Furthermore, in vitro overexpression of sirtuin3 conferred protection against hypoxia-reperfusion mediated injury by stabilizing Opa1 levels and inhibiting Drp1 protein. Two mitochondrial proteases are involved with Opa1 proteolysis. Oma1 is a stress-induced mitochondrial AAA-protease and mediates Opa1 proteolysis during stress in an ATP-independent manner [74,75,76,77]. Interestingly, Oma1-mediated Opa1 proteolysis is increased during renal IRI leading to mitochondrial fragmentation and renal injury [68]. Gene knock out of *Oma1* in mice inhibited Opa1 proteolysis and prevented mitochondrial fragmentation following renal IRI, which mitigated renal injury [68]. These results suggest that fission molecules are activated and fusion molecules are inhibited during renal IRI—this imbalance of the fission and fusion processes leads to mitochondrial fragmentation and renal injury. 

A limited number of studies are available that discuss changes in mitochondrial dynamics during renal CS/Tx. It has been shown that renal CS increases podocyte injury with a hallmark of decreased cytoplasmic density and increased round and swollen mitochondria [78]. Interestingly, renal proximal tubular epithelial cells showed mitochondrial swelling along with loss of inner mitochondrial membrane and cristae after 24 h of CS exposure [79]. Accordingly, CS/Tx induces a profound fragmentation of mitochondria [80]. An analysis of fission/fusion markers in whole-cell homogenates of rat kidneys revealed that the levels of Drp1 were reduced during CS, and were greatly reduced after CS/Tx [80]. In addition, levels of mitochondrial fission factor, a primary Drp1 receptor protein, were also reduced following CS/Tx [80]. This was surprising given that warm IRI increases localization of phosphorylated Drp1 on outer mitochondrial membrane. Interestingly, rat transplants with no CS exposure (autotransplants) did not show change in Drp1 levels [80]. However, a discrepancy on total Drp1 levels between the rat CS/Tx model and that without CS exposure (autotransplants) implicates that the CS-mediated events of reduced Drp1 levels correlate with severe mitochondrial injury. Interestingly, a recent report revealed that CS and CS/Tx induces localization of phosphorylated (S616) Drp1 on the mitochondrial membrane, and this results in mitochondrial fragmentation, and subsequently tubular cell death [81]. This report further showed that the phosphorylation of Drp1 was dependent on CS-mediated activation and mitochondrial localization of protein kinase C δ (PKCδ). The authors demonstrated that inhibition of PKCδ function via pharmacological and genomic modulation reduced Drp1 phosphorylation/localization on mitochondria, and subsequently reduced mitochondrial fragmentation and improved renal function after CS/Tx [81]. Interestingly, reduced mitochondrial fusion proteins (MFN1, MFN2) and increased Opa1 proteolysis were observed after CS/Tx. Additionally, Oma1 protein expression was altered in a rat model of CS/Tx. Based on this protein alteration, the authors hypothesized that Oma1 is over-activated in this model leading to increased OPA1 proteolysis, thus resulting in significant mitochondrial fragmentation [80]. Collectively, these studies suggest that both, fission and fusion processes are disrupted after CS/Tx leading to significant mitochondrial dysfunction after CS/Tx. 

#### 1.2.2. Mitochondrial Respiratory Complex and ROS

Mitochondria play a pivotal role in the generation of energy (in the form of ATP) and cell death signaling [82]. These dynamic organelles comprise 5 active respiratory complexes localized in the inner membrane of the mitochondria and are responsible for the generation of ATP, via oxidative phosphorylation, a process that produces ROS as a byproduct (Figure 1 and [83]). Reduction in the activity of the respiratory complexes triggers an overall bioenergetics crisis and an increase in ROS resulting in cell death. Studies in rat and pig models suggest that CS alone (without transplantation) induces ROS and decreases mitochondrial respiratory complexes (I and III) function [37,84,85,86,87,88,89]. Studies further suggest that more severe mitochondrial respiratory dysfunction occurs after CS/Tx (cold ischemia + warm IRI) than transplantation without CS (only warm IRI) and further revealed that CS/Tx decreases the function of mitochondrial complexes I, II, III, and V [80]. Mitochondrial respiratory dysfunction during renal CS/Tx leads to reduced levels of ATP, accumulation of ROS, and increased renal damage, suggesting that the “damage” signals from CS likely begin with alterations in mitochondrial function [37,80,84,85,86,87,88,89,90]. Normally, mitochondrial ROS are primarily detoxified by manganese superoxide dismutase (MnSOD), a mitochondrial antioxidant enzyme. MnSOD scavenges superoxide radicals and converts them to hydrogen peroxide (Figure 1D). The hydrogen peroxide is further catalyzed by glutathione peroxidase and catalase to molecular oxygen and water (Figure 1D). IRI leads to decrease of MnSOD activity, which increases oxidative stress [91,92]. Interestingly, overexpression of the MnSOD enzyme has been shown to blunt ROS production during renal CS/rewarming, protecting against cell death in proximal tubular cells [88]. On the other hand, ischemia-mediated reduced ATP levels impair the ion exchange/transport channel with subsequent accumulation of calcium [93,94]. This activates a calcium-dependent increase in phospholipase, endonuclease, and protease, which leads to apoptosis [93,94,95]. During early IRI, mitochondrial calcium overload, oxidative stress, elevated phosphate concentrations, and adenine nucleotide depletion promote the opening of mitochondrial permeability transition pores (mPTP) in the inner mitochondrial membrane [96]. This disrupts the H+ concentration on both sides of the membrane and abolishes the ATP-synthase driving force. The opening of mPTPs also allows cytosolic molecules to freely enter into the mitochondria, resulting in mitochondrial swelling and rupture [97]. ATP depletion also leads to changes in the cytoskeleton, due to ATP being required for actin polymerization [98]. The lack of actin polymerization results in a loss of brush border and cell-cell contact, resulting in cellular detachment [99]. 

Cardiolipin, a tetra-acyl anionic phospholipid, participates in the maintenance of structural integrity of the mitochondrial membrane. It is localized at the highest level in the inner mitochondrial membrane and helps to organize cristae structures [100], stabilize mitochondria respiratory complexes and/or supercomplexes, facilitate the optimal transfer of electrons, and optimize bioenergetics efficiency [101,102,103]. It has been demonstrated that cardiolipin works as a seal on ATP synthase (mitochondrial complex V) during electron transfer and ATP production [104,105]. Interestingly, the major components of phospholipids, including cardiolipin, are reduced in mitochondrial fractions of ischemic hearts [106], and further IRI leads to oxidative modification of cardiolipin in adult hearts [107]. Wiswedel et al. showed that ROS in the presence of cytochrome c induces cytochrome c peroxidase activity, which catalyzes cardiolipin peroxidation, resulting in mitochondrial dysfunction [108]. Cardiolipin peroxidation leads to the release of cytochrome c from the inner mitochondrial membrane and promotes its release into the cytosol via mPTP-dependent and -independent mechanisms [109,110]. On a different note cardiolipin inhibits platelet-activating factor [111] and therefore, potentially has great significance in solid organ transplantation because the existence of anticardiolipin antibodies may be a risk factor for renal thrombotic microangiopathy, which may be associated with acute graft dysfunction [112]. All of these studies suggest that cardiolipin plays a critical role in maintaining mitochondrial function and cellular bioenergetics and serves as an attractive drug target in renal transplantation.

#### 1.2.3. Mitophagy

Eukaryotic cells have a well-developed mechanism to remove damaged cytoplasmic proteins or organelles via a process called autophagy. Autophagy involves the lysosome-dependent degradation of damaged cytoplasmic components sequestered by autophagosomes [113]. Though there are three forms of autophagy, including chaperone-mediated autophagy, macroautophagy, and microautophagy; macroautophagy is the best studied and is what we refer to here as autophagy. Autophagy is induced during stress (e.g., nutrient deprivation) as a protective mechanism, where it clears cytotoxic protein aggregates and dysfunctional organelles in a multi-step fashion and plays an adaptive role in cell survival. Although autophagy is regarded as a non-selective process, increasing lines of investigation suggest that eukaryotic cells harbor selective autophagy processes, through which damaged organelles, such as mitochondria, are removed from the cells [114]. When mitochondria are damaged, a selective autophagy process, also known as mitophagy is induced [114,115]. Mitophagy is induced during stress as a protective mechanism that clears dysfunctional mitochondria in a multi-step fashion and plays an adaptive role in cell survival. Normally, fission process assists in separating damaged mitochondrial content from healthy ones. Mitophagy involves the lysosome-dependent degradation of the damaged mitochondria sequestered by autophagosomes. Mitophagy is an adaptive response to IRI-mediated stress and promotes cell survival during IRI [72,116,117,118,119,120,121,122]. There is no reported evidence on the role of the mitophagy process during renal CS/Tx. Sporadic reports are available on autophagy, but there are no conclusive data on this process during renal CS/Tx [123,124]. In a mouse model of renal transplantation, inhibiting autophagy increased apoptotic cell death both in vitro CS/rewarming and in vivo after and CS/Tx [124]. One caveat for this study is that kidneys were stored in heparinized saline instead of a standard CS solution (e.g., Viaspan). Another study using a pig renal CS/Tx model showed that despite an increase in autophagy markers following xenon treatment during CS, early graft (renal) function was not preserved [123]. Further, it is not clear whether autophagy was induced after CS/Tx (without xenon treatment). To our knowledge, no other studies have evaluated the role of autophagy during CS-mediated renal injury, and it is clear that more effort is needed to carefully examine the role of autophagy during CS/Tx. Mitophagy is a dynamic and complicated process, and the current tools to investigate this dynamic autophagic flux remains very challenging [113,125,126]. Given the extent of graft damage and dysfunction, it is anticipated that the autophagic flux is interrupted during extended CS with transplantation. However, thorough and succinct research is required to define the status and the role of mitophagy in the context of protein homeostasis and graft dysfunction after renal CS/Tx. Given that mitophagy is essential in clearing damaged mitochondria, if this process is interrupted then the injured mitochondrial contents will be released into the cytosol which could lead to apoptotic/necrotic injury. 

Injured mitochondria are also the source of damage-associated molecular patterns (DAMP), which promote inflammation during diseases [127,128]. Recently, mitochondria-derived DAMPs have been shown to activate pathways that lead to alterations in the immune system and organ function. Mitochondrial damage releases mitochondrial components (e.g., mtDNA and/or mtRNA, cytochrome c, succinate, and ATP), which are recognized as potential DAMPs promoting inflammation. For example, increased levels of mtDNA were found in the urine of critically ill septic patients with acute kidney injury [129], and elevated mtDNA in urine was associated with kidney injury in an animal model of sepsis [130]. Interestingly, urinary mtDNA levels were found to be significantly higher in patients with delayed graft function and in cases of deceased donor transplantation [131]. This study further showed that the subjects with acute rejection had higher urinary mtDNA levels than those without abnormalities. CS/Tx is associated with renal parenchymal inflammation; however, it remains to be demonstrated if there is a causal relationship between mitochondrial DAMPs and inflammation during CS/Tx. Increasing the removal of damaged mitochondria by mitophagy has several positive effects, such as removing DAMPs and decreasing inflammation [132,133,134,135]. Thus, activating mitophagy is an appealing therapeutic strategy during diseases, such as CS/Tx, which may assure mitochondrial quality control and improve renal function following CS/Tx. 

### 1.3. The Proteasome

**Figure 2:**
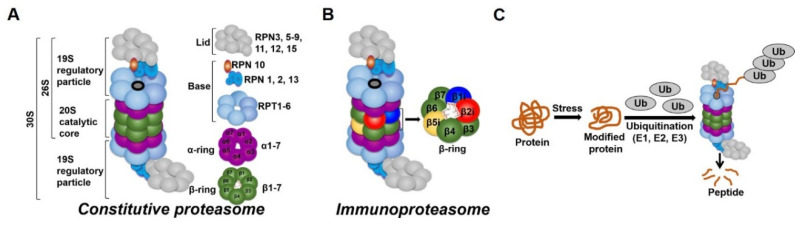
Ubiquitin-proteasome system (UPS). (**A**) The constitutive proteasome (26S or 36S) is a barrel-shaped organelle that is made up of 20S catalytic core and one (26S) or two (30S) 19S particle(s). The 20S catalytic core is made up of stacks of two β rings (β1–β7) and two α rings (α1–α7). The 19S regulatory particle is made up of a lid and a base with multiple subunits. (**B**) The immunoproteasome is a proteasome variant that is normally found in immune cell compartments. However, in response to inflammation, constitutive proteasome subunits (β1, β2, and β5) are exchanged for the immunoproteasome subunits (β1i, β2i, and β5i) in most non-immune cells in the body. (**C**) Damaged or modified proteins are ubiquitinated (with the concerted action of ubiquitinating enzymes), which is then recognized by the constitutive proteasome for degradation. The constitutive proteasome selectively degrades ubiquitinated proteins to small peptides (**A**); it has 3 to 7 protease active sites (β-catalytic subunits) that hydrolyze peptide bonds in a chymotrypsin (β5 subunit)-, trypsin (β2 subunit)-, or caspase (β1 subunit)-like fashion. Following protein degradation, the peptides are released and recycled.

In all tissues, proteasomes are crucial for degrading modified, misfolded, and damaged proteins. The constitutive proteasome, selectively degrades ubiquitinated proteins (via the concerted actions of ubiquitinating enzymes) to small peptides (Figure 2A and [136,137,138,139]). The constitutive proteasome, a multi-subunit holoenzyme of ~2.5 MDa, is made up of two distinct sub-domains, namely, a 20S catalytic core particle and 1 or 2 19S regulatory particle(s) (Figure 2A). The 20S core particle is a barrel-shaped complex, composed of stacks of 2 β-rings (each ring made up of β1-7 subunits) in the center and 2 α-rings (each ring made up of α 1-7 subunits) on each end (Figure 2A). The α-rings (subunits) appear to have a regulatory function, allowing only unfolded substrates to enter into the 20S catalytic core. The β-ring of the 20S proteasome has 3 to 7 active sites (β-catalytic subunits) (Figure 2A) that hydrolyze peptide bonds in a chymotrypsin-like (β5 subunit), trypsin-like (β2 subunit), or caspase-like (β1 subunit) fashion (Figure 2C and [140]). The 19S regulatory particle recognizes and unfolds the ubiquitinated substrates before allowing the substrate to enter the 20S pore [141]. Functionally, the 19S particle is divided into a base and a lid. The base consists of an ATPase ring, made up of 6 AAA-ATPase subunits (Rpt1-6), and 3 non-ATPase subunits (Rpn1, 2, and 13) (Figure 2A). The ATPase subunits consume ATP to unfold the substrate and help translocate it to the pore of the 20S catalytic core. The lid, which is linked to the base by the Rpn10 subunit (Figure 2A), assists in the efficient degradation of the ubiquitinated substrates. The immunoproteasome is a proteasome variant that is normally found in immune cell compartments [142]. However, in response to inflammation, catalytic subunits (β1, β2, and β5) of the constitutive proteasome are exchanged for immunoproteasome subunits (β1i, β2i, and β5i) in most non-immune cells (Figure 2B and [142,143]).

**Figure 3:**
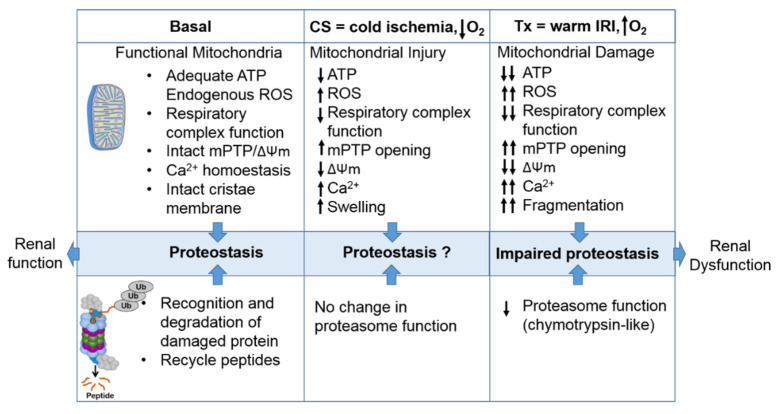
The intricate relationship between the mitochondria and proteasome. Schematic summary depicting mitochondrial and proteasomal changes during cold storage (CS) and transplantation. During CS, mitochondrial respiration function, ATP level, and mitochondrial membrane potential (ΔΨm) decreases, whereas ROS and calcium levels increase leading to an increase of mitochondrial permeability transition pore (mPTP) opening. This leads to increased swelling and decreased function of mitochondria. These changes are further exacerbated and sustained following transplantation leading to mitochondrial fragmentation and bioenergetic crisis. Proteasome function remains unchanged during renal CS, whereas the chymotrypsin-like proteasome function is decreased following transplantation. This leads to alteration of mitochondrial protein homeostasis and acute tubular necrosis after transplantation and significantly decreases renal function.

The proteasome maintains functional protein homeostasis, also known as proteostasis, by monitoring misfolded and damaged proteins; however, this is a challenge in the context of renal CS/Tx, especially in kidneys that have undergone prolonged CS (Figure 3). Given that prolonged CS followed by warm IRI produces ROS [37,39], and that ROS modulate the constitutive proteasome function [144,145,146], we can postulate that CS/Tx-mediated ROS could trigger denaturation of intracellular proteins and modulation of the constitutive proteasome function. Indeed, a recent report demonstrated that the chymotrypsin-like activity of the proteasome was compromised after renal CS/Tx [147], and that this correlated with severe renal dysfunction [80,90]. A study performed by using pharmacological inhibition of chymotrypsin-like activity of the proteasome during warm IRI showed aggravated renal damage [148]. Genetic or pharmacologic modulation of proteasome function (chymotrypsin-like) inhibition, achieved by siRNA or bortezomib treatment, respectively, in rat proximal tubular cells showed increase of ROS production [149]. At this point, the mechanisms of proteasome dysfunction during CS/Tx are not known. One of the possible mechanisms could be CS/Tx-mediated post-translational modification of the proteasome subunits because this mechanism has been described to modulate proteasome function and assembly in various experimental models [150,151]. 

It is well-accepted that CS/Tx produces inflammation and releases inflammatory cytokines [152]. Immunoproteasomes are induced and activated in response to the inflammatory cytokines, and help in processing donor-derived antigen effectively. Unlike the constitutive proteasome, the immunoproteasome is resistant to oxidative stress and can function in an ATP-independent manner [153,154]. For example, interferon-induced ROS activate the immunoproteasome [154]. There are a handful of transplant studies showing a negative correlation of immunoproteasome activity with organ function. In this context, a recent report by Li et al. indicates that pharmacological inhibition of the immunoproteasome with ONX 0914 (a reversible β5i inhibitor) reduces donor-specific antibody production in a rat model of renal CS/Tx [155]. Thus, it is worth investigating whether ONX 0914 should be included in the CS solution. This is particularly exciting because some studies indicate that ONX 0914 protects against cardiac and neuronal IRI [156,157]. However, one caveat of the Li et al. study is that the authors used a very short CS time (~35 min) that is likely not clinically relevant. Future studies with longer CS times are needed to verify whether blunting the immunoproteasome with ONX 0914 during CS confers protection after transplantation. The specific mechanisms underlying compromised proteasome function and exacerbated immunoproteasome activity during renal CS/Tx are not understood and should be addressed.

Although the proteasome manages protein turnover, aberrations in the expression and function of the constitutive proteasome and immunoproteasome are implicated in the pathogenesis of several human diseases, including cancer, autoimmune disorders, and inflammatory diseases [142,143,158,159,160,161]. In the context of CS/Tx, it is clear that the initial damage occurs within the epithelial or vascular compartments within the kidneys (during cold ischemia) that eventually triggers an immune response following blood reperfusion (warm IRI) after CS/Tx. It is expected that prolonged CS followed by transplantation disrupts protein homeostasis, which overwhelms the immune response, and this could directly impact long-term graft and patient outcomes. 

### 1.4. Interactions between the Mitochondria and Proteasome and the Potential Role of this Interaction during CS/Tx

An intricate relationship exists in all tissues between the mitochondria and the proteasome [162]. The proteasome pathway helps to remove damaged mitochondrial proteins [163,164]. As previously mentioned, mitochondrion are unique organelles having their own DNA that synthesizes 13 different proteins (subunits of the mitochondrial respiratory complexes), while the remaining mitochondrial proteins are encoded by the nuclear genome and synthesized in the cytosol, after which they are imported into mitochondria. During this process, the damaged or misfolded mitochondrial proteins must be degraded before their import into mitochondria [162]. It appears that the UPS plays a key role in ubiquitinating and degrading the modified mitochondrial proteins. A study using a transgenic mouse model that expressed tagged ubiquitin in the heart showed that mitochondrial proteins comprised the largest group of the ubiquitinated proteins and accounted for 38% of all identified proteins [165]. Interestingly, these identified mitochondrial proteins were found from all mitochondrial locations, namely, the outer membrane, inner membrane, intermembrane space, and matrix. In another study performed in yeast, genes related to the 26S proteasome were found to be among the most essential genes that are required to shape mitochondrial morphology [166]. The imported mitochondrial proteins are constantly exposed to ROS, a byproduct of oxidative phosphorylation (Figure 1 and [83]), which may lead to modification of these proteins. These damaged or modified mitochondrial proteins must be removed from the mitochondria. Mitochondrial quality control systems include mitochondrial proteases, mitophagy, and the UPS and these systems help to clear the damaged, unfolded, or misfolded proteins from mitochondria. The proteasome is an integral component of the mitochondrial protein quality control system and mediates the degradation of damaged proteins via the mitochondria associated degradation system, also known as outer mitochondrial membrane-associated degradation system [163,167,168,169,170]. Xu et al. showed that p97/VDC, an AAA-ATPase, retro-translocates Mcl1, an outer membrane mitochondrial protein, and delivers it to the cytosolic proteasome for degradation [171]. Several other reports support the notion that the p97/VDC mediates extraction of damaged mitochondrial proteins and delivers them to the cytosolic UPS for degradation [167,172,173]. On the other hand, ATP and ROS are considered critical determinants for the constitutive proteasome function [174,175,176]. Normally, the 19S regulatory particle associates with the 20S proteasome in the presence of ATP to permit entry of substrate via gate opening into the 20S catalytic core [177,178,179,180]. Moreover, the constitutive proteasomes require ATP to unfold substrate and carry out proteolytic function; mitochondria are the supplier of this ATP. During renal CS, the activity of the mitochondrial respiratory complexes (I, II, and III) decreases, which compromises ATP production [80,90]. In addition to an impairment of ATP production, ischemic injury also induces the catalysis of ATP during CS [23]. IRI process further depletes the ATP level along with impairment of mitochondrial respiratory activity during CS/Tx [90]. Mitochondrial dysfunction not only depletes ATP but also increases mitochondrial ROS following CS and transplantation [84,88,89,181,182]. It has been shown that, mild oxidative stress dissociates the 20S catalytic core from the 19S regulatory particles [145,146,183] (Figure 2B). Importantly, the dissociated 20S core does not require ATP and ubiquitin tag, and thus, can degrade proteins in an unregulated manner [184,185]. In contrast, higher levels of ROS are shown to inhibit the UPS [145,146,183,184,185,186], suggesting a complex role for ROS as a modulator of UPS activity. Interestingly, a pre-clinical model shows that the constitutive proteasome function is not compromised during prolonged renal CS, but the chymotrypsin-like proteasome activity is compromised following CS/Tx [147], suggesting a possible link between mitochondrial dysfunction (ATP depletion and increased ROS) and compromised proteasome function. It also appears that mitochondrial injury precedes proteasome dysfunction during renal CS/Tx. Proteasome dysfunction further exacerbates mitochondrial dysfunction after transplantation. This demonstrates the delicate relationship between these two organelles and further implicates that any shift in the balanced interactions could disrupt protein homeostasis and result in renal cell death/tissue damage. We believe that the balanced function of these organelles is necessary to maintain proteostasis and overcome stress during renal CS/Tx. 

## 2. Therapeutics Targeting Mitochondria to Improve Outcome after Transplantation

**Figure 4:**
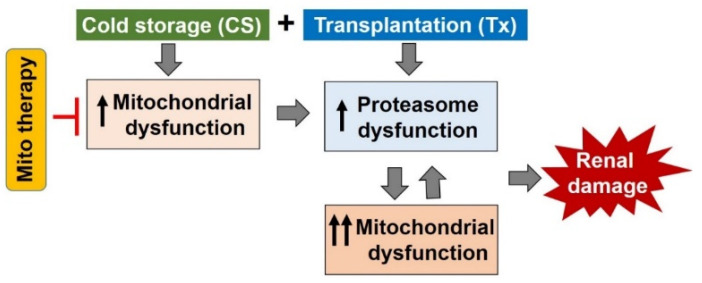
Targeting therapy during cold storage to improve renal outcomes after transplantation. Schematic illustration of a potential therapeutic strategy to improve renal function after transplantation (modified with permission from [147]). It is hypothesized that mitochondrial therapy should be introduced during cold storage (CS) to blunt mitochondrial injury early on. This may prevent proteasome as well as mitochondrial damage/dysfunction after transplantation, and therefore, may improve renal outcomes.

The maintenance of proteome integrity is extremely important for renal cell viability during stressful conditions, especially during CS/Tx. Based on the discussed findings, CS/Tx appears to alter mitochondrial and proteasome (constitutive proteasome and immunoproteasome) function (Figure 3), which culminate to amplify inflammation and renal damage during CS/Tx. Designing therapeutics for mitochondria that maintain the delicate balance with the proteasome will be a winning strategy for improving graft outcome after renal CS/Tx (Figure 4). The most recent therapeutic approaches to and studies of post-transplantation renal injury are based on the assumption that reperfusion introduces a burst of renal damage. As highlighted above, it seems clear that mitochondrial dysfunction occurs early during renal CS (within 4 h) [90]. It is important to outline that targeting mitochondria early during CS and preserving proteasome and mitochondrial function might represent a critical point for CS outcomes following transplantation (Figure 4). Several mitochondrial therapeutics have been tested pre-clinically that successfully ameliorate renal damage in various diseases. Sadly, few mitochondrial therapeutics have been tested to improve renal transplant outcomes; it is anticipated that this area of research will increase significantly in the future. Here we will discuss potential mitochondrial therapeutics that have been tested experimentally in various renal diseases that may provide beneficial effects during renal CS/Tx. 

ROS induce numerous cellular pathways and it is well accepted that ROS production is accelerated during renal CS/Tx. Mitochondrial ROS is produced early during CS (4 h). The mitochondria-targeted antioxidant, MitoQ, has shown a promising effect in blunting CS-mediated mitochondrial ROS production, preventing respiratory complex dysfunction, and ameliorating renal injury in ex vivo experiments [84,89]; however, this protective effect needs to be evaluated with in vivo experiments of CS/Tx. The rationale for this compound’s clinical applicability is supported by pre-clinical studies in rodent models that show a protective effect of MitoQ during IRI [187,188]. As discussed earlier, Drp1 is an important molecule that regulates mitochondrial fission, and reducing fission by inhibiting Drp1 conferred protection in an animal model of IRI [66]. In case of CS/Tx, inhibition of Drp1 phosphorylation in mice, as achieved by pharmacological and genomic modulation of upstream kinase pathway, CDCδ, reduced mitochondrial fragmentation and improved renal function [81]. These observations, in both warm and cold IRI models, are unprecedented and appear to be in contrast with the proposed theory that increased fission process during stress removes damaged mitochondria and then confers protection against stress during disease condition. Similarly, Opa1 proteolysis by activated Oma1 results in decrease of fusion process. Targeting Oma1 activity by genetic modulation of Oma1 reduced mitochondrial swelling/fragmentation and improved IRI-mediated renal function [68]. These results indicate that IRI (cold and warm) exhibits severe alteration of mitochondrial dynamics favoring increased mitochondrial fission/fragmentation. Since CS/Tx models exhibit fragmented mitochondria, it is anticipated that targeting fission (e.g., Drp1) and fusion (e.g., Oma1) during renal CS may provide a beneficial effect on renal transplant outcomes. However, these targeted approaches are expected to create a balance between fission and fusion processes by favoring fusion process to complement normal mitochondrial morphology/function. 

Another component of the increased oxidative stress involved in acute kidney injury (e.g., IRI) is that it leads to peroxidation of cardiolipin. Cardiolipin plays an important role in the conformation of the mitochondrial cristae and the correct assembly of mitochondrial super complexes for proper mitochondrial respiratory function [189]. Therefore, cardiolipin has been recognized as an important mitochondrial drug target during acute kidney injury [71,190,191]. SS 31, a mitochondria targeted tripeptide, discovered by the Szeto group [192], has been shown to bind to cardiolipin with high affinity forming a complex that inhibits cytochrome c oxidase activity by protecting its heme component [190,193]. Studies using SS 31 have demonstrated that it protected cristae membranes, prevented mitochondrial swelling, restored ATP levels, mitigated tubular cell death, and improved renal function during renal IRI ([71,194]. Currently, this compound is under clinical trial investigating its efficacy in ameliorating IRI post-angioplasty for renal arterial stenosis [195]. Results from the pilot trial study produced encouraging results with improved renal function following renal arterial stenosis [195]. 

Oxidative stress, cardiolipin peroxidation, and calcium overload all lead to increased mPTP opening [96]. The persistent increase of mPTP opening leads to mitochondrial swelling and rupture of the outer mitochondrial membrane, which then leads to necrotic or apoptotic cell death [196,197,198]. Inhibition of the mPTP opening has been shown to ameliorate renal IRI and acute kidney injury. The widely used clinical immunosuppressive drug, Cyclosporin A (CSA), is a potent inhibitor of the mPTP [199]. The use of CSA in a rat model has shown some double-sided effects—the low dose seems to improve renal function, histopathologic damage, and antioxidant enzyme status in rats with renal IRI [200], but the high dose of CSA appeared to increase fission [201], decrease mitochondrial respiratory function [202], and induce nephrotoxicity [203]. Nevertheless, a correct dose of CSA is still worth investigating due to the importance of mPTP in the pathogenesis of renal CS/Tx. Regardless, preserving mitochondrial function during CS and CS/Tx is an ultimate goal and it appears that mitochondrial ROS is upstream to the above discussed pathological consequences. Therefore, blunting mitochondrial ROS and improving mitochondrial function during CS would render normalization of numerous cellular pathways including mitochondrial dynamics, cardiolipin peroxidation, and mPTP opening. Likewise, restoring normal mitochondrial function during CS would implicate the restoration of proteasomal function following transplantation, which ultimately would improve renal outcome after CS/Tx.

## 3. Summary

Reducing the renal CS-mediated injury could help better utilize donor kidneys, prevent graft failure, improve long-term graft survival, and decrease the mortality rate of patients with ESKD. Understanding the molecular mechanisms underlying CS-related renal damage may lead to the development of new therapies that improve graft outcomes following CS/Tx. A thorough and careful examination of CS-related pathways including mitochondria and proteasomes may facilitate the identification of novel targets. For instance, future studies are warranted to identify mechanisms of alteration of mitochondrial and proteasome dysfunction during CS and CS/Tx. This could serve as a basis for therapeutic interventions to mitigate mitochondrial injury during cold preservation, which would help in preventing proteasome alteration and ultimately in ameliorating renal damage following CS/Tx.
